# Polyorchidism: An Up-to-Date Systematic Review

**DOI:** 10.3390/jcm12020649

**Published:** 2023-01-13

**Authors:** Krzysztof Balawender, Agata Wawrzyniak, Józef Kobos, Michał Golberg, Andrzej Żytkowski, Michał Zarzecki, Jerzy Walocha, Michał Bonczar, Andrzej Dubrowski, Agata Mazurek, Edward Clarke, Michał Polguj, Grzegorz Wysiadecki, Anna Smędra

**Affiliations:** 1 Department of Normal and Clinical Anatomy, Institute of Medical Sciences, Medical College of Rzeszow University, 35-315 Rzeszów, Poland; 2 Department of Histology and Embryology, Institute of Medical Sciences, Medical College of Rzeszow University, 35-315 Rzeszów, Poland; 3 Department of Histology and Embryology, Chair of Anatomy and Histology, Medical University of Lodz, 90-752 Łódź, Poland; 4 Norbert Barlicki Memorial Teaching Hospital No. 1, Medical University of Lodz, 90-001 Łódź, Poland; 5 Department of Polish Dialectology and Logopedics, Faculty of Philology, University of Lodz, 90-236 Łódź, Poland; 6 Department of Anatomy, Jagiellonian University Medical College, 33-332 Kraków, Poland; 7 Department of Normal and Clinical Anatomy, Chair of Anatomy and Histology, Medical University of Lodz, 90-752 Łódź, Poland; 8 Chair and Department of Forensic Medicine, Medical Faculty, Medical University of Lodz, 90-001 Łódź, Poland

**Keywords:** anatomical anomalies, gonads, polyorchidism, supernumerary testis, urology, urogenital system

## Abstract

Polyorchidism is a rare male urogenital tract anomaly characterized by at least one supernumerary testis in the scrotum or ectopically. According to data based on our systematic review, 76% of the supernumerary testes (SNTs) were located in the scrotum, and 24% were extra-scrotal (*p* < 0.001). Among testes located outside the scrotum, 87% were found in the inguinal canal and 13% in the abdominal cavity. In 80% of cases, the diagnosis of SNT was made based on imaging tests, and the remaining 20% of cases were detected incidentally during surgery. The imaging tests performed (US or MRI) resulted in a significantly higher rate of patients who qualified for observation vs. surgical treatment (45% vs. 35%, *p* < 0.001). The most common conditions associated with SNT were ipsilateral inguinal hernia (15% of cases) and cryptorchidism (15% of cases). Surgery (orchidopexy/orchidectomy) was performed on 54% of patients with SNT, and the decision to observe the SNT was made in a total of 46% of patients (*p* = 0.001). The therapeutic approach depends on the location of the SNT and the presence of factors that raise suspicion of neoplastic proliferation.

## 1. Introduction

Polyorchidism is a rare male urogenital tract anomaly characterized by at least one supernumerary testis (SNT) situated in the scrotum or ectopically. Despite the awareness of this defect for more than 120 years [1], there is still a lack of consensus between clinicians regarding the management of this condition. So far, robust data that could help create an algorithm for clinical decision support while detecting polyorchidism is still lacking. The cases described to date confirm the problematic nature of the anomaly both in terms of diagnosis (various locations of SNT and difficult differential diagnosis, e.g., problems with distinguishing it from tumor masses) and therapy (surgical intervention versus observation).

Embryologically, polyorchidism can result from an accidental division of the germinal ridge before eight weeks of gestation. Different levels at which the transverse genital ridge division may occur reflect the diverse types of the supernumerary testes, taking into account that the different variants are characterized by the presence or absence of extra vas deferens and epididymis [2].

In the present analysis, the polyorchidism classification proposed by Bergholz et al. [3] was applied, and bilobed testis cases (incomplete polyorchidism) were treated as a separate type of SNT. Based on the classification of Bergholz et al. [3], a testis drained by a deferent duct is known as type A, while all other cases are referred to as type B. Type A testes can be further divided into subgroups according to the attached structures as follows: A1, the drained SNT has its own epididymis and vas deferens; A2, the drained SNT can have its own epididymis, but shares a common deferent duct with its neighbor (i.e., the properly developed ipsilateral testis); and A3, the drained SNT can share a common epididymis (and duct) with its neighbor. Type B testes can also be divided into subgroups according to the attached epididymis as follows: B1, the undrained SNT has its own epididymis; B2, the undrained SNT does not have its own epididymis and consists only of testicular tissue.

This review aims to analyze and evaluate the applied diagnostic and therapeutic interventions for cases of polyorchidism described in the last two decades. The available literature needs an up-to-date systematic analysis of polyorchidism cases to propose an algorithm for managing this rare defect.

## 2. Materials and Methods

### 2.1. Search Strategy

A systematic literature search was conducted by two authors independently, using major electronic medical databases (Science Direct, Web of Science, and PubMed) up to August 2021, for articles that could potentially be used in this systematic review. The search strategy used in this study included the following terms: “polyorchidism” and “supernumerary testis”.

**Figure 1:**
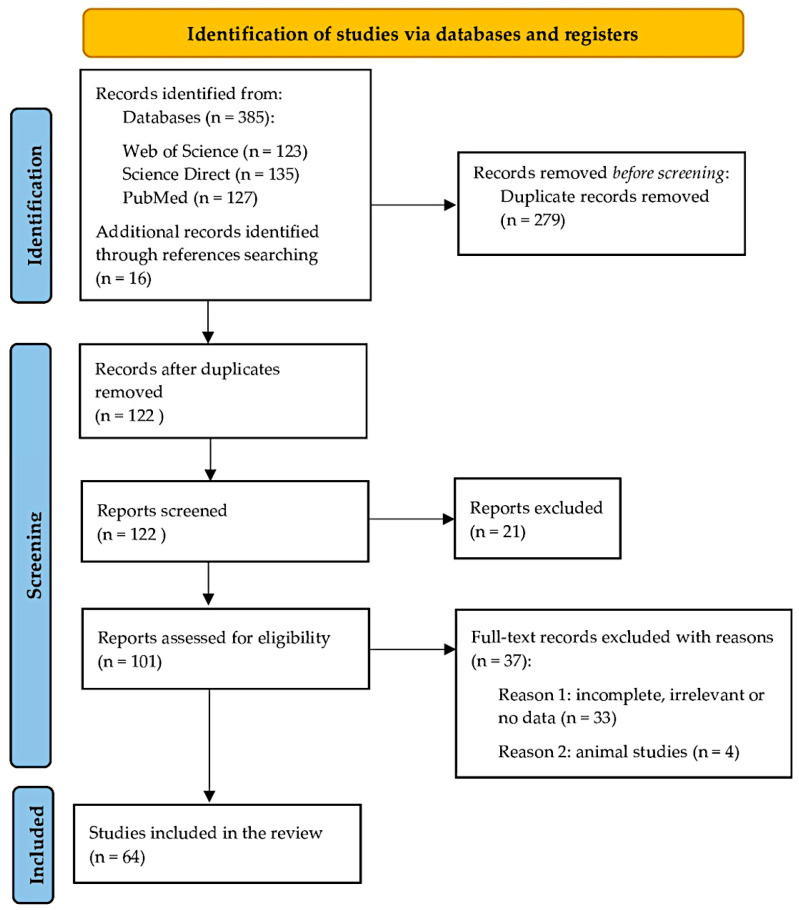
The PRISMA flow diagram.

The number of records found in the PubMed database for polyorchidism/SNT for the years 1931–2021 was 231. After initially examining the publications, it was decided to narrow the time frame of the literature search to the last two decades. Therefore, the publication period included the past 22 years (2000–2021). The primary reason for this is the lack of data in early publications was due to the inability to perform high-quality ultrasound (US) scanning and magnetic resonance imaging (MRI). Following acquisition of the full texts, a reference search was carried out to identify other potentially eligible articles that could have been missed in the electronic database search. The Preferred Reporting Items for Systematic Reviews and Meta-analyses [PRISMA] guidelines were followed while conducting this study [4] (Figure 1).

### 2.2. Eligibility Assessment

Study selection and data extraction were divided between two authors to allow for an independent double-check of articles and data. We applied the following exclusion criteria:

- Review articles and conference abstracts;
- Incomplete or impossible to extract data;
- Or research performed on animals.

Since there were many studies that involved patients with concomitant pathologies (i.e., inguinal hernia, cryptorchidism, hydrocele, testicular tumor, testicular torsion, male infertility), it was decided that articles reporting the presence of the SNT during such interventions will be included in this systematic review.

Help from medical professionals who were fluent in both English and the language of the publication was sought in times when the authors lacked fluency in the latter. Any inconsistencies within the included works were solved by contacting the authors of the original studies. Whenever the information could not be obtained, all reviewers participated in the assessment until a consensus was reached.

### 2.3. Data Extraction

Data extraction of all studies that met the inclusion criteria for this systematic review was carried out by two reviewers individually. Data on the modality of the studies, sample size, and anatomical variants of the SNT were obtained. The authors of the original studies were contacted for clarification or additional information whenever any discrepancy was found in the reported research.

### 2.4. Study Endpoints

The analysis paid particular attention to the type of SNT according to the Bergholz et al. classification (details of classification in the Introduction) [5], the location of the SNT (scrotal or ectopic), and the presence of more than one SNT. The secondary endpoint addressed the relationship between the presence of SNT and comorbidities (cryptorchidism, hernia, testicular torsion, hydrocele, testicular tumors) and the use of imaging studies in differential diagnosis. The tertiary endpoint was the evaluation of the treatment used for the diagnosis of SNT (surgery versus observation) and the type of surgery (orchidopexy versus orchiectomy).

### 2.5. Quality Assessment

The AQUA tool was used to estimate the quality and reliability of the included studies. Five domains were evaluated in the analysis: Objective(s) and Subject Characteristics, Study Design, Methodology Characterization, Descriptive Anatomy, and Reporting of Results. Each domain was evaluated as having a “Low”, “High”, or “Unclear” risk of bias [6].

### 2.6. Statistical Analysis

The Chi^2^ test and regression analysis (linear and quadratic) were used to analyze the synthesized data in this study. The data for the model were selected based on the experience of the researchers. The R Studio program was used to calculate the results (RStudio Team (2020), Integrated Development for R. RStudio, PBC, Boston, MA URL http://www.rstudio.com/, 1 August 2022). Statistical significance was set with a *p*-value < 0.05. Since the obtained data came from case reports or case series, meta-analytical evaluation was not performed.

## 3. Results

### 3.1. Study Identification

The study identification process is summarized in Figure 1. In the preliminary search, 385 articles were found in the preliminary search that could meet the inclusion criteria. Further, 16 studies were identified during the reference search. A total of 279 studies were duplicates which were excluded. After the initial screening of abstracts and titles, 21 records were considered ineligible: the majority were reviews or reported irrelevant data. One hundred and one articles were subject to full-text analysis, and finally, 64 studies were included in this systematic review.

### 3.2. Characteristics of the Included Studies

The characteristics of the included studies are summarized in Table 1. Sixty-four patients were evaluated in this systematic review. The research included in this study had a published date range of 2000–2021. After initially examining the publications, the decision was made to narrow the time frame of the literature search to the last two decades. The primary reason for this is the lack of data in early publications due to the inability to perform high-quality ultrasound (US) scanning and magnetic resonance imaging (MRI). The scope of searched literature was worldwide. Since not all patients had both US and MRI imaging studies performed, individual cases were selectively chosen for analysis.

### 3.3. Quality Assessment

The AQUA tool evaluation is summarized in Table 2. Overall, the vast majority of studies were evaluated as having a “High” risk of bias in Objective(s) and Subject Characteristics and Methodology Characterization due to the lack of complete information on the baseline characteristics and demographics of the patients, as well as the specialty and experience of the scientists in charge of a particular part of the study. Domains: Study Design and Reporting of Results were evaluated as having a “Low” risk of bias for all included studies.

### 3.4. Prevalence of Polyorchidism and Localization of the SNT

About 76% of the SNTs were located in the scrotum, and 24% were extra-scrotal; (*p* < 0.001). Among testes located outside the scrotum, 87% were found in the inguinal canal and 13% in the abdominal cavity. In 65% of the cases, SNT occurred on the left side (*p* < 0.001).

### 3.5. Types of Polyorchidism

According to the classification by Bergholz et al., the most common subtype of SNT was type A2 (38%), followed by type A1 (26%) and type A3 (16%). Type B accounted for 10% of SNTs (B1-6%, B2-4%), and bilobed testes accounted for 10% of all SNTs.

### 3.6. Radiological Examinations of the SNT

In 80% of cases, the diagnosis of SNT was made based on radiological examinations, and the remaining 20% of cases were detected incidentally during surgery. The radiological examinations such as US and/or MRI resulted in a significantly higher rate of patients qualifying for observation vs. surgical treatment (45% vs. 35%, *p* < 0.001). The US examinations helped qualify STN patients for observation vs. surgical treatment (in 45% vs. 33%, *p* < 0.001; Table 3). MRIs also allowed SNT patients to qualify for observation vs. surgical treatment (in 33% vs. 13%, *p* < 0.001; Table 4).

### 3.7. Conditions and Symptoms Associated with Polyorchidism

The most common conditions associated with SNT were ipsilateral inguinal hernia (15% of cases) and cryptorchidism (15% of cases). Testis hydrocele was found in 7% of the cases, and testicular torsion in 7%. In 4% of the cases, cancer was diagnosed in the SNT (seminoma and intratubular germ cell neoplasia (IGCN). 31% of patients with SNT reported scrotal pain. 35% of patients were diagnosed with SNT incidentally during examinations for unrelated conditions (inguinal hernia, hydrocele, testicular torsion). Among the 65 SNT cases analyzed, only nine patients underwent semen analysis (in this group, five patients had semen analysis abnormalities-low sperm concentration, abnormal sperm morphology, and decreased sperm motility).

### 3.8. Size of the SNT

**Figure 2:**
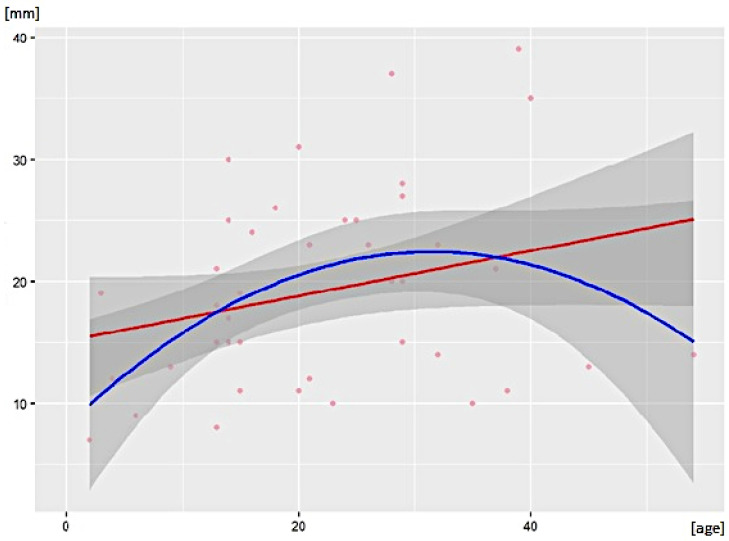
Linear (red curve) and quadratic (blue curve) effects of age on the score level of the variable “supernumerary testis length [mm]”.

The mean size in the long axis of the SNT was 19.9 mm (SD ± 7.47 mm). The analysis included 42 surveyed observations; the remaining reports selected for analysis did not have data on the dimensions of the SNT. Quadratic regression analysis showed a significant prediction (F [2, 39] = 4.07; *p* < 0.05). Analysis of the R-squared coefficient values showed that the regression model of the included independent variables (age, age-squared) explained about 17% (13% after correction) of the variation in SNT length (Table 5). The correlation coefficients were R2 = 0.17 and adj. R2 = 0.13, respectively. These coefficients indicate that the quadratic model provides a better explanation of the variation in SNT length in the long axis (Figure 2). The number of significant predictors in the model was 2. The analysis showed that the mean level of the SNT length variable was 8.11 mm.

### 3.9. Type of Treatment

Surgery (orchidopexy/orchidectomy) was performed on 54% of patients with SNT, and the decision to observe the SNT was made in 46% of patients (*p* = 0.001). For the group of patients with SNT in the scrotum, 59% were referred to observation, while 41% underwent surgical intervention (*p* = 0.001). In the group of patients with ectopic SNT, 92% underwent surgery, and 8% were qualified for observation (*p* = 0.001).

Among the patients who underwent surgery, 45% underwent orchidopexy, while 55% underwent orchidectomy. In cases with SNT located in the scrotum, orchidopexy was performed in 59% of patients, while orchidectomy was performed in 41% of patients (*p* = 0.071; Table 6). In the ectopic SNT group, 75% had orchidectomy, and 25% underwent orchidopexy (*p* = 0.071; Table 6).

### 3.10. Tetraorchidism and Pentaorchidism

There were nine cases of tetraorchidism (2 SNTs) and two cases of pentaorchidism (3 SNTs) in the study group. In eight patients, the SNTs were located in the scrotum (89%). The most common types of tetraorchidism were A2, A3, and B2 based on the Bergholz et al. classification (25% of each type), and seven patients had bilateral SNTs. Tetraorchidism was histologically confirmed in 44% of patients, while all patients underwent the US, and 56% of them underwent MRI. Among the tetra/pentaorchidism cases, no neoplasms were found; 22% of patients were diagnosed with cryptorchidism, 11% with hydrocele, and 33% with testicular torsion. One-third of those cases were detected incidentally, and 44% of patients reported scrotal pain. 56% of patients with tetraorchidism qualified for observation, 22% underwent orchidopexy, and 22% underwent orchidectomy.

## 4. Discussion

Polyorchidism is a very rare developmental anomaly of the male reproductive system. The knowledge of embryology, clinical manifestations, and diagnostic and therapeutic pathways for SNT is based on single case reports and one meta-analysis published in 2009 [3]. The current analysis aimed to update the knowledge on polyorchidism—considering contemporary medical imaging methods—and attempts to create a diagnostic and therapeutic consensus in detecting an isoechoic/isodense tissue mass located in the scrotum or ectopically. 80% of all SNT cases were testes that had developed a deferent duct (separate or shared with the ipsilateral testis, depending on the type), and 76% were located in the scrotum. Considering a supernumerary testis’s 50–65% reproductive potential [9,67], this makes a compelling case for testicular preservation.

The etiology of SNT still needs to be fully understood. Articles published to date point to a transverse division of the genital ridge before the eighth week of gestation as the most likely theory for the formation of SNTs [2,11,26,68]. The different levels at which the transverse genital ridge division may occur reflect the diverse types of SNTs, bearing in mind that the presence or absence of an extra ductus deferens and epididymis can characterize individual variants. A distinct and extremely rare form of polyorchidism is the bilobed testis, which most likely results from an incomplete division of the germinal ridge [16,23,64]. This variant accounted for 10% of all SNT cases in the present analysis.

Polyorchidism cases are typically detected incidentally in men presenting with comorbid conditions or in patients concerned with discovering an extra mass in the scrotum. The most common comorbidities included ipsilateral inguinal hernia and cryptorchidism. Despite a lack of clinical manifestation in many cases, one-third of SNT patients reported scrotal pain. In young SNT patients with associated pain, testicular torsion should always be considered in the differential diagnosis. The risk of testicular torsion in the general population is 0.025% [66], while for cryptorchidism, it is 0.25% [63]. In the current analysis, 7% of patients with SNT were diagnosed with torsion (a 280-fold higher absolute risk compared to the general population). Bergholz et al. [3] reported that up to 15% of SNT patients are diagnosed with torsion of one of the testes on the ipsilateral side. 

Polyorchidism is associated with an increased risk of testicular cancer. Given that the estimated risk of testicular cancer in the general population is 0.006% [65], the existence of SNT increases this risk. In our study, the prevalence of testicular malignancy was 4%, while Bergholz et al. [3] reported a rate of 5.7%. An additional factor that increases this risk is an ectopic SNT (cryptorchidism increases the risk of testicular cancer by 2.2 to 4.7 compared to the general population [69,70]). Due to the relatively low incidence of cancers associated with SNT, it is not easy to assess which histological types are the most common. For the cases included in our analysis, histopathological examinations revealed seminoma and intratubular germ cell neoplasia (IGCN) [24,59]. Bergholz et al. [3] also reported choriocarcinoma, teratoma, and embryonal carcinoma cases.

Modern imaging radiological techniques for identifying paratesticular masses include high-resolution US and MRI. Today, owing to their widespread availability, high sensitivity, and specificity, these methods should be the examinations of the first choice for the differential diagnosis of paratesticular masses. In our analysis, in patients diagnosed between 2000 and 2021, up to 80% had a US, and 50% had an MRI (in a previous analysis, only 27% of patients had a US or an MRI [3], which, for obvious reasons, was due to the limited availability of the equipment and the poorer quality of equipment at the time). An alternative to MRI for diagnosing a scrotal mass may be a contrast-enhanced ultrasound (CEUS), performed after intravenous administration of ultrasound contrast agents consisting of microbubbles. CEUS is a safe method well tolerated by patients. This technique has been used to evaluate scrotal lesions and produces a better visualization of vascularity than the color Doppler technique (it can detect microvessels as small as 2–7 µm) [39].

The primary purpose of radiological diagnostic techniques imaging when suspecting an SNT is to rule out neoplastic lesions and reduce surgical intervention rates when the SNT is structurally normal. To the best of our knowledge, the current study is the first to have statistically evaluated the effects of radiological techniques during the diagnostic procedures for the SNT. Radiological techniques such as US and/or MRI resulted in significantly more patients being qualified for observation versus surgical intervention (45% vs. 35%). When considering only the group of patients with an SNT diagnosed using MRI, up to 72% were qualified for observation. According to the European Society of Urogenital Radiology (ESRU), MRI imaging of normal adult testes shows T2 hyper- and T1 hypo-to-isointense, homogenous oval structures with diffusion restriction, surrounded by T2/T1 hypointense tunica albuginea. The signal intensity of an SNT is almost identical to that of an anatomically normal testis [39].

Moreover, the imaging examinations allowed for an analysis of the size of SNTs. The current study used a mathematical model that demonstrated that the size of the SNT depends on the patient’s age and is subject to the same variability observed for anatomically normal testes [10,71]. The length of the SNT, measured in the long axis, increases with the development of the body until about 25 years of age and, subsequently, undergoes a gradual involution, which is observed in older males (Figure 2).

The management of polyorchidism has evolved over time and is still under debate. As polyorchidism is an extremely rare congenital abnormality, there are a few cases to facilitate evidence-based recommendations. Traditional management involves surgical removal of the SNT due to the increased risk of malignancy. Histological examination of surgically explored cases revealed functional parenchyma in 50–65% of SNTs [67]. Thus, patients with a scrotal SNT without radiological indications of malignancy may be recommended for conservative management and monitoring, with regular self-examination, clinical examinations by health professionals, and non-invasive imaging (US or MRI). In our study, 59% of patients with an SNT in the scrotum were qualified for observation, whereas, in the surgical intervention group, 59% had orchidopexy. These results indicate a testicular preservation trend in urology reflected by significantly more frequent qualification for observation or orchidopexy when an SNT is detected in the scrotum. A separate issue in the case of intent to preserve the SNT is the assessment of the presence of pathways releasing sperm count of the SNT. The differentiation of SNTs based on Bergholz et al.’s classification [3] into type A or type B is possible with MRI or intraoperative observation. In polyorchidism with an extra-scrotal testis, especially in a young patient of reproductive age, orchidopexy is recommended if feasible, followed by observation. If an SNT is associated with any signs of malignancy, radical orchidectomy is recommended.

**Figure 3:**
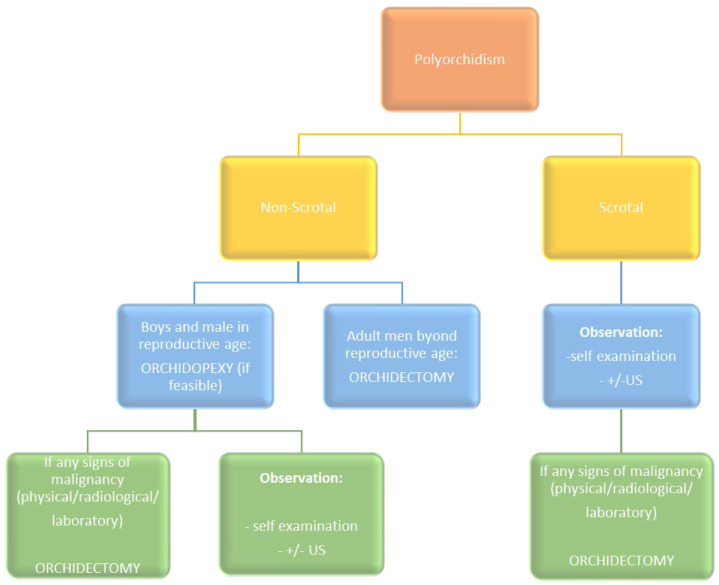
Proposed therapeutic algorithm for polyorchidism. Adopted from Balawender et al. [61] under Creative Commons Attribution-Non Commercial (unported, v3.0) License.

Here, we propose a therapeutic algorithm based on the current knowledge that can be used in everyday clinical practice (Figure 3). The resulting algorithm is particularly applicable to the diagnosis of triorchidism. Detecting two or more SNTs requires a personalized therapeutic approach for each case, depending on the location and the progression of the SNTs (tetraorchidism and pentaorchidism accounted for 13% and 3% of patients, respectively, in our study).

### Limitations

Due to the casuistic nature of the issue, our systematic review is based on an analysis of case reports or case series; the lack of broader studies in previous publications did not allow us to conduct a meta-analysis. After analyzing the collected publications, the authors decided to apply the time criterion to the selection of the literature (publications from the period 2000–2021 were analyzed). The primary reason for this is the lack of data in early publications due to the inability to perform high-quality ultrasound (US) scanning and magnetic resonance imaging (MRI).

## 5. Conclusions

Polyorchidism is a rare anomaly of the male reproductive system; however, the differential diagnosis of paratesticular masses should always consider the possible presence of an SNT. Current results show a trend toward testicular preservation after SNT diagnoses. Contemporary diagnostic imaging should include a mandatory US and an MRI of the abdomen and scrotum. The therapeutic approach depends on the location of the SNT and the presence of factors that raise suspicion of neoplastic proliferation.

## Figures and Tables

**Table 1:** Characteristics of the included studies. SNT—supernumerary testis; US—ultrasound; MRI—magnetic resonance imaging; S—scrotum; E—ectopic; R—right; L—left; Y—yes; N—no.

	Year of Publ.	Age	# of SNT	SNT Localization	SNT Laterality	US	MRI	Treatment Options		
								Observation	Orchidopexy	Orchidectomy
Mandalia [7]	2020	13	1	S	L	Y	Y	Y	-	-
Chowdhary [8]	2016	1	1	E	L	N	N	-	Y	-
Alamsahebpour [9]	2013	13	2	S	L & R	Y	N	Y		-
Ibrahim [10]	2016	13	2	S	L & R	Y	N	-	Y	-
Vijayanadh [11]	2009	37	1	E	R	Y	Y	Y	-	-
Leodoro [12]	2014	2	1	S	R	Y	N	-	Y	-
Haley [13]	2008	1	1	E	R	N	N	-	-	Y
Repetto [14]	2010	2	2	S	L	Y	Y	Y	-	
Arslanoglu [15]	2012	20	1	S	L	Y	Y	-	-	Y
Méndez-Gallart [16]	2012	4	1	S	L	Y	N	-	-	Y
Sarma [17]	2008	16	1	S	L	Y	N	Y	-	
Bergholz [5]	2007	6	1	E	R	N	N	-	-	Y
Ersin [18]	2006	21	1	S	L	Y	Y	Y	-	-
Ferro [19]	2005	12	1	S	L	Y	N	-	Y	-
Spranger [20]	2002	23	2	S	L & R	Y	Y	Y	-	-
Tigabie [21]	2020	4	1	E	L	Y	N	-	-	Y
Bayissa [22]	2020	43	1	E	R	N	N	-	-	Y
Cohen [23]	2017	1	2	S	L & R	Y	N	-	Y	-
Boussaffa [24]	2018	41	1	S	R	N	N	-	-	Y
Myers [25]	2017	14	3	S	2L & R	Y	Y	Y	-	-
Kumar [26]	2008	3	1	E	L	Y	Y	-	Y	-
Abduljabbar [27]	2015	25	1	S	L	Y	Y	Y	-	-
Aldughiman [28]	2020	14	1	S	L	Y	Y	Y	-	-
Ojaghzadeh [29]	2020	18	1	S	R	Y	Y	Y	-	-
Moghadam [30]	2020	1	3	E	2L & R	Y	N	-	Y	-
Fonseca-Sosa [31]	2019	32	1	E	R	Y	N	-	Y	-
Nepal [32]	2019	21	1	S	R	Y	Y	-	Y	-
Özman [33]	2018	29	1	S	L	Y	Y	Y	-	-
Kealey [34]	2018	29	1	S	L	Y	N	-	Y	-
Ben Lustig [35]	2017	2	1	E	L	N	N	-	Y	-
Gune [36]	2021	28	1	S	L	Y	N	Y	-	-
Di Cosmo [37]	2016	45	1	S	R	Y	N	-	-	Y
Bhandarwar [38]	2016	24	1	E	L	N	N	-	-	Y
Rafailidis [39]	2017	15	1	S	L	Y	Y	-	-	-
Uğuz [1]	2016	20	2	S & E	L & R	Y	Y	-	-	Y
Balasar [40]	2017	4	1	E	L	N	N	-	-	Y
Celik [41]	2014	28	1	S	L	Y	Y	Y	-	Y
Nayak [42]	2011	20	1	S	L	N	N	-	Y	-
Arlen [43]	2014	14	1	S	L	Y	N	-	Y	-
Jakhere [44]	2014	52	1	S	L	Y	Y	Y	-	-
Belba [45]	2014	54	1	S	L	Y	Y	Y	-	-
Sağlam [46]	2013	14	1	S	L	Y	Y	Y	-	-
Lawrentschuk [2]	2004	15	1	S	R	Y	N	-	Y	-
Chintamani [47]	2009	13	1	S	L	Y	Y	Y	-	-
Hassan [48]	2008	32	1	S	R	Y	Y	Y	-	-
Khedis [49]	2008	47	2	S	L & R	Y	N	-	-	Y
Rajbabu [50]	2007	37	1	S	L	Y	N	Y	-	-
Bhogal [51]	2007	15	1	S	R	Y	Y	Y	-	-
Nane [52]	2007	29	1	S	L	Y	Y	-	-	Y
Deveci [53]	2004	26	1	S	L	Y	Y	Y	-	-
Danrad [54]	2004	9	1	S	L	Y	Y	Y	-	-
Roessingh [55]	2003	14	1	S	R	N	N	-	Y	-
Spranger [20]	2002	23	1	S	L & R	Y	Y	Y	-	-
Chung [56]	2001	35	2	S	L	Y	Y	Y	-	-
Schafer [57]	2018	38	1	S	L	Y	N	Y	-	-
Duymuş [58]	2016	40	2	S	L	Y	Y	Y	-	-
Ghose [59]	2007	39	1	E	R	Y	Y	-	-	Y
Topsakal [60]	2011	20	1	S	R	Y	Y	-	Y	-
Balawender [61]	2021	29	1	E	R	Y	N	-	-	Y
Nikolic [62]	2021	28	1	E	R	Y	Y	-	-	Y
Beiko [63]	2010	13	1	S	L	Y	N	-	Y	-
De Carli [64]	2009	3	1	S	L	Y	N	Y	-	-
Haffar [65]	2021	39	1	S	L	Y	N	Y	-	-
Halliday [66]	2013	12	1	S	L	Y	N	Y	-	-

**Table 2:** The risk of bias analysis based on the AQUA tool evaluation.

Study		Risk of Bias				
		Objective(s) and Subject Characteristics	Study Design	Methodology Characterisation	Descriptive Anatomy	Reporting of Results
Mandalia [7]	2020	High	Low	High	Low	Low
Chowdhary [8]	2016	High	Low	High	Low	Low
Alamsahebpour [9]	2013	High	Low	High	Low	Low
Ibrahim [10]	2016	High	Low	High	Low	Low
Vijayanadh [11]	2009	High	Low	High	Low	Low
Leodoro [12]	2014	High	Low	High	High	Low
Haley [13]	2008	High	High	High	Low	Low
Repetto [14]	2010	High	Low	High	Low	Low
Arslanoglu [15]	2012	Low	Low	High	Low	Low
Méndez-Gallart [16]	2012	Low	Low	High	Low	Low
Sarma [17]	2008	High	Low	High	Low	Low
Bergholz [5]	2007	High	Low	High	Low	Low
Ersin [18]	2006	High	High	Low	High	Low
Ferro [19]	2005	High	Low	High	Low	Low
Spranger [20]	2002	High	Low	High	Low	Low
Tigabie [21]	2020	High	Low	High	Low	Low
Bayissa [22]	2020	Low	Low	High	Low	Low
Cohen [23]	2017	High	Low	High	High	Low
Boussaffa [24]	2018	High	Low	Low	Low	Low
Myers [25]	2017	High	High	High	Low	Low
Kumar [26]	2008	High	Low	High	Low	Low
Abduljabbar [27]	2015	High	Low	High	Low	Low
Aldughiman [28]	2020	High	Low	High	Low	Low
Ojaghzadeh [29]	2020	High	Low	High	Low	Low
Moghadam [30]	2020	High	Low	High	Low	Low
Fonseca-Sosa [31]	2019	High	Low	High	Low	Low
Nepal [32]	2019	High	Low	High	Low	Low
Özman [33]	2018	Low	Low	High	Low	Low
Kealey [34]	2018	High	Low	High	Low	Low
Ben Lustig [35]	2017	High	Low	High	Low	Low
Gune [36]	2021	High	Low	High	Low	Low
Di Cosmo [37]	2016	High	Low	High	Low	Low
Bhandarwar [38]	2016	High	Low	High	High	Low
Rafailidis [39]	2017	Low	Low	High	Low	Low
Uğuz [1]	2016	High	Low	High	Low	Low
Balasar [40]	2017	High	Low	High	Low	Low
Celik [41]	2014	High	Low	High	Low	Low
Nayak [42]	2011	High	Low	High	Low	Low
Arlen [43]	2014	High	Low	Low	Low	Low
Jakhere [44]	2014	High	Low	Low	Low	Low
Belba [45]	2014	High	Low	High	Low	Low
Sağlam [46]	2013	High	Low	High	High	Low
Lawrentschuk [2]	2004	High	Low	High	Low	Low
Chintamani [47]	2009	High	Low	High	Low	Low
Hassan [48]	2008	High	Low	High	Low	Low
Khedis [49]	2008	High	Low	High	Low	Low
Rajbabu [50]	2007	High	Low	High	Low	Low
Bhogal [51]	2007	High	Low	High	Low	Low
Nane [52]	2007	High	Low	High	Low	Low
Deveci [53]	2004	High	Low	High	Low	Low
Danrad [54]	2004	High	Low	High	Low	Low
Roessingh [55]	2003	High	Low	High	Low	Low
Spranger [20]	2002	High	Low	High	Low	Low
Chung [56]	2001	High	Low	High	Low	Low
Schafer [57]	2018	High	Low	High	Low	Low
Duymuş [58]	2016	High	Low	High	Low	Low
Ghose [59]	2007	High	Low	Low	Low	Low
Topsakal [60]	2011	Low	Low	High	Low	Low
Balawender [61]	2021	High	Low	High	Low	Low
Nikolic [62]	2021	High	Low	High	Low	Low
Beiko [63]	2010	High	Low	High	High	Low
De Carli [64]	2009	High	Low	High	Low	Low
Haffar [65]	2021	High	Low	High	Low	Low
Halliday [66]	2013	High	Low	High	Low	Low

**Table 3:** Management of supernumerary testis in a group of patients with ultrasound performed in the course of diagnosis.

SNT Position	Surgery	Observation	Chi^2^ Test *p*-Value
Scrotal, no. of patients (%)	18 (33)	25 (45)	<0.001 df 1
Non-scrotal, no. of patients (%)	12 (22)	0 (0)	

**Table 4:** Management of supernumerary testis in a group of patients with magnetic resonance imaging of the scrotum/abdomen performed in the course of diagnosis.

SNT Position	Surgical Treatment	Observation	Chi^2^ Test *p*-Value
Scrotum, no. of patients (%)	4 (13)	11 (33)	<0.001 df 1
Ectopic, no. of patients (%)	13 (42)	4 (13)	

**Table 5:** Linear and quadratic influence of age on the level of variable results—the supernumerary testis length. B = non-standardized regression coefficient; s.e. = standard error for B; *p* = statistical significance.

	B	s.e.	*p*
Variables in the linear regression model			
Constans	15.11	2.59	<0.001
Age	0.18	0.10	<0.10
Variables in the quadratic regression model			
Constans	8.11	4.07	<0.10
Age	0.91	0.35	<0.05
Age-squared	-0.01	0.01	<0.05

**Table 6:** Surgical interventions for supernumerary testis depending on the location.

SNT Position	Orchidopexy	Orchidectomy	Chi^2^ Test *p*-Value
Scrotal, no. of patients (%)	10 (59)	7 (41)	0.071
Non-scrotal, no. of patients (%)	3 (25)	9 (75)	

## Data Availability

All the data is available within the study. This process can be initiated upon request to the corresponding author.
